# *Rickettsia sibirica mongolitimonae* in Traveler from Egypt

**DOI:** 10.3201/eid1609.100258

**Published:** 2010-09

**Authors:** Cristina Socolovschi, Sébastien Barbarot, Maeva Lefebvre, Philippe Parola, Didier Raoult

**Affiliations:** Author affiliations: Université de la Méditerranée, Marseille, France (C. Socolovschi, P. Parola, D. Raoult);; Centre Hospitalier Universitaire de Nantes, Nantes, France (S. Barbarot, M. Lefebvre)

**Keywords:** Rickettsia sibirica mongolitimonae, zoonoses, vector-borne infections, ticks, Egypt, travel, lymphangitis-associated rickettsiosis, letter

**To the Editor:** Tick-borne rickettsioses are zoonoses caused by spotted fever group (SFG) *Rickettsia* spp (*1*), which have been reported as a frequent cause of fever in international travelers (*2*). In Egypt, Mediterranean spotted fever caused by *Rickettsia conorii* transmitted by the brown dog tick, *Rhipicephalus sanguineus,* is known to be present, although cases are rarely documented. Moreover, an emerging pathogen, *R. aeschlimannii,* has been detected in *Hyalomma dromedarii* ticks, collected from camels, and in *H. impeltatum* and *H. marginatum rufipes,* collected from cows (*3*). We report a case of *Rickettsia sibirica mongolitimonae* infection in a French traveler who returned from Egypt

In September 2009, a previously healthy 52-year-old man living in France was admitted to the infectious diseases unit of a hospital in Nantes, France, with a 10-day history of fever, asthenia, headache, and arthromyalgia. Three days earlier, he had returned from a 2-week trip to Egypt. He had fever (38°C), painful axillary lymphadenopathies, and an inoculation eschar surrounded by an inflammatory halo on the left scapular area (Figure A1), but he did not have a rash. During his travel, he had been unsuccessfully treated for headache, arthromyalgia, and diarrhea by amoxicillin-clavulanate (3 g/d), nonsteroidal antiinflammatory drugs, and gentamicin cream on the eschar for 3 d. No tick bite was reported by the patient. We suspected an SFG rickettsiosis. The patient received 200 mg doxycycline in a single dose and rapidly improved.

The immunofluorescence assay for antibodies reactive against SFG antigens showed increased levels of immunoglobulin M (titer 16) and G (titer 128). Results of Western blot with cross-adsorption assays supported the hypothesis that the infection was caused by *R. sibirica mongolitimonae* (*1*). To identify the involved rickettsiae, PCR amplifications and sequencing *glt*A, *omp*A, and *omp*B fragment genes of *Rickettsia* spp. and multispacer typing (MST), based on the sequence of variable intergenic spacers, were performed by using DNA samples obtained from an eschar biopsy and a lesion swab (*4*,*5*). A negative control (sterile water and DNA from a sterile biopsy specimen) and a positive control (DNA from *R. montanensis*) were included in each test. Amplicon sequencing confirmed the presence of *R. sibirica mongolitimonae* DNA in patient samples. The sequence homology to *R. sibirica mongolitimonae* DNA was *omp*A, 99.4%; *glt*A, 99.7%; and *omp*B, 100% (GenBank accession nos. DQ097082, DQ097081, and AF123715, respectively). The MST sequences were 100% homologous to the genotype of *R. sibirica mongolitimonae* MST type U (idem HA-91). We injected shell vial cultures with eschar biopsy specimens (*4*). Fifteen days later, positive Gimenez staining and immunofluorescence confirmed the presence of *Rickettsia* sp. in cell culture, and *R. sibirica mongolitimonae* was identified by PCR and sequencing as described above (Figure A1).

*R. sibirica mongolitimonae* was first isolated in Beijing in 1991 from *H. asiaticum* ticks (formerly named *R. sibirica* HA-91), and the first human infection was reported in 1996 (*4*). Since that time, *R. sibirica mongolitimonae* infections have been diagnosed in 15 additional patients: 12 from Europe (France, Portugal, Greece, and Spain) and 3 from Africa (Algeria, South Africa, and the present patient who returned from Egypt). The application of genotypic criteria to *R. sibirica mongolitimonae* classified the organism as a subspecies of *R. sibirica* group, in spite of its distinct serotypes and specific epidemiologic features compared to *R. sibirica sibirica*, the causative agent of Siberian tick typhus or North Asian tick typhus (*1*).

*R. sibirica mongolitimonae* causes lymphangitis-associated rickettsiosis. The available clinical features for the only 16 reported cases (10 men, 6 women) include fever in all patients (range 38˚C–39.5°C), chills (3/16 patients), headache (13/16), myalgia (13/16), arthralgia (3/16), cutaneous rash (11/16), enlarged lymph nodes (10/16), lymphangitis expanding from an inoculation eschar to the draining node (6/16), and retinal vasculitis in a pregnant woman (*6*,*7*). Two patients exhibited 2 eschars. Most eschars were on the legs, but some patients had an eschar on the back, the abdomen, the arm, or the face. The patients’ median age was 50 years (range 20–76 years). A tick bite or tick handling was reported for 5 patients, but no tick was collected for further examination. In France, 7 patients probably came in contact with *R. sibirica mongolitimonae*–infected ticks in their gardens, and 2 other patients were probably exposed during a walk in the Camargue National Park, where migratory birds are frequently present (*7*). Infection with *R. sibirica mongolitimonae* occurred primarily between March and September. A single case was reported in December in Greece. *R. sibirica mongolitimonae* has been detected in several *Hyalomma* spp. ticks in Niger, Greece, the People’s Republic of China, Senegal, and in *Rh. pusillus* ticks in Portugal (*6*–*8*). Although *Hyalomma* spp. ticks seem to be associated with *R. sibirica mongolitimonae*, more experimental data are needed to determine the tick vectors and reservoirs of this rickettsia.

Clinicians in Egypt and those who may see patients returning from this country should be aware that several species of rickettsiae are found in this region. Thus, they should consider a range of SFG rickettsial diseases in the differential diagnosis of patients with febrile illnesses.

## Figures and Tables

**Figure A1:**
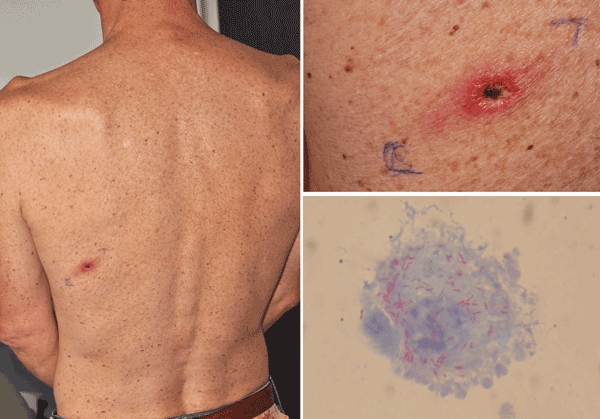
A) Eschar on the scapular area of a patient infected with *Rickettsia sibirica mongolitimonae*. B) Close-up view of eschar. C) *R. sibirica mongolitimonae* growing in L929 cells, stained in red by Gimenez staining.
